# Enhanced chondrogenesis from human embryonic stem cells

**DOI:** 10.1016/j.scr.2019.101497

**Published:** 2019-08

**Authors:** Tao Wang, Puwapong Nimkingratana, Christopher A. Smith, Aixin Cheng, Timothy E. Hardingham, Susan J. Kimber

**Affiliations:** a Faculty of Biology, Medicine and Health, University of Manchester, UK; b Wellcome Centre for Cell-Matrix Research, Faculty of Biology, Medicine and Health, University of Manchester, Oxford Road, Manchester M13 9PL, UK

## Abstract

Human embryonic stem cells (hESCs) have great potential for the repair of damaged articular cartilage. We developed a serum-free 14-day protocol for hESC differentiation into chondrocyte progenitors, which surprisingly lacked strong cartilage matrix production in in vitro tests. In order to direct these progenitors to a more mature phenotype, we investigated substituting different members of the TGFβ family in the protocol. Initially, we supplemented, or substituted GDF5 (day 11–14), with combinations of BMP7 and TGFβ-1, or −3, but these modifications yielded no improvement in matrix gene expression. However, replacing BMP4 with BMP2 (days 3–10 of the protocol) resulted in a more rapid increase in SOX9 gene expression and increased expression of chondrogenic genes SOX5, ACAN and COL2A1. The replacement of BMP4 with BMP2 also enhanced the formation of chondrogenic cell aggregates, with greater deposition of type II collagen. This change was not accompanied by hypertrophic chondrocyte marker COL10A1 expression. The results demonstrate that BMP2 has greater specificity for the generation of chondrogenic cells from hESCs than BMP4 and this was consistent in two hESC lines (HUES1 and MAN7). hESC-chondrogenic cells derived with either BMP2 or BMP4 were tested in vivo by implanting them in fibrin into osteochondral defects in the femur of RNU rats. Repaired cartilage tissue, positive for Safranin O and type II collagen was detected at 6 and 12 weeks with both cell sources, but the BMP2 cells scored higher for tissue quality (Pineda score). Therefore, BMP2 is more effective at driving chondrogenic differentiation from human pluripotent stem cells than BMP4 and the effect on the resulting chondroprogenitors is sustained in an in vivo setting.

## Highlights

• BMP2 stimulates increased chondrogenic gene expression from hESC chondroprogenitors compared to BMP4.
• Other TGFΒ members were not effective.
• BMP2 promoted COL2a1 positive aggregate formation. with no effect on culture expansion or apoptosis.

## Graphical abstract

**Figure:**
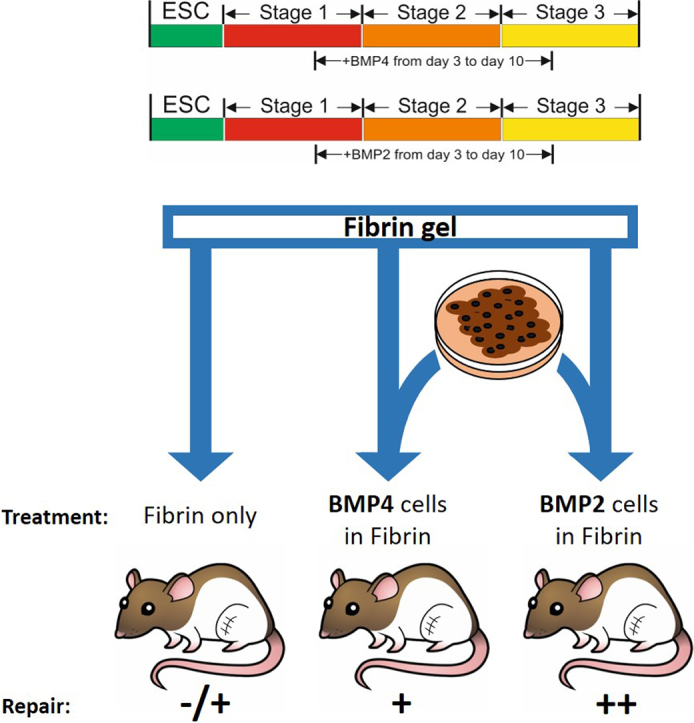

## 1 Introduction

Hyaline cartilage forms the load-bearing surface of articular joints and is required for friction-free movement. The tissue is avascular and aneural and is composed primarily of an extracellular matrix rich in type II collagen and proteoglycans. It is maintained by a single cell type-the chondrocyte, which occupy <3% of the tissue volume. Articular cartilage is mechanically important in the joint, but it is vulnerable to damage though acute injury, or during joint disease. Partly due to its avascular nature, it has poor intrinsic capacity for repair, which predisposes the joint to developing osteoarthritis (OA). An important clinical aim is thus to repair focal defects and eventually larger lesions caused by the degeneration of the cartilage during OA. This requires strategies to replace damaged areas with new cartilage and the most promising of these strategies is cell-based treatments with donor cells. Autologous chondrocyte implantation (ACI) was developed to treat focal cartilage defects (Brittberg et al., 1994; Filardo et al., 2012), but complications such as chondrocyte hypertrophy resulting in vascular invasion and calcification have impeded progress (Hettrich et al., 2008; Pelttari et al., 2006). Moreover, the need for 2 operations and invasive harvesting of intact cartilage, together with de-differentiation of cultured chondrocytes during monolayer expansion, has hindered wider application (Kang et al., 2007). There is thus limited evidence that this approach can provide a permanent or large-scale solution. Stem cells, which can respond to developmental signals to create chondrocytes, are an alternative source of cells. Mesenchymal stem cells (MSCs), such as from human bone marrow, can be induced to form chondrocytes (Pittenger et al., 1999) and have been used for cartilage repair (Wakitani et al., 2011), but they have limited capacity for expansion as a bulk supply of cells (Stolzing et al., 2008). Human pluripotent stem cells (hPSC) in contrast can undergo unlimited expansion and can differentiate into any cell type in the body (pluripotency). This offers the potential to generate chondrocytes for the treatment of cartilage repair (Cheng et al., 2014a). Thus, hPSCs offer an alternative source of cells for allogeneic cell-based cartilage repair. Induced pluripotent stem cells (iPSCs) derived from adult somatic cells provide the opportunity to generate joint disease models and, in due course, may also form a source of therapeutic cells.

Our lab previously developed a directed differentiation protocol for hESCs, which exploits normal developmental signals to generate chondrogenic cells with high efficiency and purity (Cheng et al., 2014a; Oldershaw et al., 2010). To activate lateral plate and chondrogenic mesodermal induction we used BMP4, a growth factor shown through mouse knockout studies (Winnier et al., 1995) to be essential for murine mesoderm formation and implicated in mesenchymal condensation and the generation of skeletal elements in limb bud development (Bandyopadhyay et al., 2006; Tsumaki et al., 2002). Using our protocol we showed that implanted hESC-derived chondroprogenitors could repair an osteochondral defect in immunocompromised Nude rats (Cheng et al., 2014b). Although these chondroprogenitor cells formed cartilage in vivo they responded poorly to the in vitro conditions under which adult bone marrow MSCs formed cartilage matrix in 3D cell cultures. From these results we concluded that this protocol does not proceed through an adult MSC-like stage, but generates cells resembling embryonic limb bud chondrogenic mesenchyme (Griffiths, Ronshaugen and Kimber unpublished). Hence, we refer to these cells as chondroprogenitors. We therefore set out to investigate ways to direct these cells to a more mature chondrocyte phenotype with enhanced production of extracellular matrix.

BMP2, BMP4, and BMP7, as well as GDF5, are all expressed in the joint and thought to play roles in its development and the formation of cartilage (Francis-West et al., 1999; Gaissmaier et al., 2008; Hatakeyama et al., 2004; Zou et al., 1997). BMP4 has been used extensively to drive diverse mesoderm specification from hESCs (Laflamme et al., 2007; Xu et al., 2002; Zhang et al., 2008) and is used routinely in chondrogenic differentiation (Diekman et al., 2012; Lee et al., 2013; Craft et al., 2015). However, BMP2, also a known chondrogenic factor (Bandyopadhyay et al., 2006), has been employed in some studies in the differentiation of hESCs to chondrocytes with promising results (Yamashita et al., 2015) as have other TGFβ-family growth factors (Nakagawa et al., 2009). We therefore investigated the effects of substituting, or adding other TGFβ-family growth factors, which are expressed during cartilage development in vivo and have reported pro-chondrogenic properties. We proposed that these alternative growth factors might enhance the chondrogenic phenotype and boost expression of extracellular matrix genes.

## 2 Materials and methods

### 2.1 Cell culture and directed chondrogenic differentiation

**Fig. 1:**
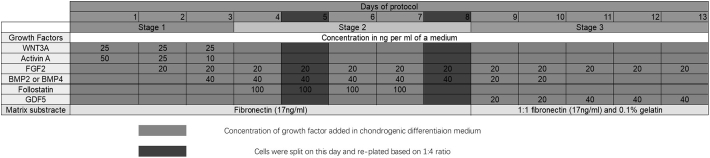
Summary timeline of the protocol for chondrogenic differentiation of hESCs using BMP2 or BMP4.

HUES1 and MAN7 human embryonic stem cell lines were cultured as previously described (Cheng et al., 2014b; Oldershaw et al., 2010). Briefly, hESCs were cultured on mouse embryonic fibroblasts (iMEFs, mitomycin C inactivated), at 6 × 10^4^/cm^2^ on 0.1% gelatin (Sigma) coated plates in Dulbecco's modified Eagle's medium (DMEM) supplemented with 20% (v/v) knockout serum replacement, 2 mM l-glutamine, 1% (v/v) nonessential amino acids (NEAA), 0.1 mM β-mercaptoethanol, 1% (v/v) penicillin/streptomycin (all Invitrogen) and 10 ng/ml FGF2 (Autogen Bioclear, Wiltshire, UK). Cells were passaged using TrypLE™ (Invitrogen). For differentiation cells were transferred for at least 7 days to chemically defined feeder-free culture (Baxter et al., 2009). Cells were lifted from the iMEF layers with TrypLE™, and plated onto fibronectin-coated (Millipore) tissue culture flasks with feeder-free medium: 50:50 F12:DMEM (Lonza) supplemented with l-glutamine, 1% NEAA, 0.1 mM β-mercaptoethanol, 1% penicillin/streptomycin, 0.1% bovine serum albumin (BSA) (Sigma), 1% (vol/vol) N2 supplement 2% (vol/vol), B27 supplement (both Invitrogen), 10 ng/ml activin A, 4 ng/ml neurotrophin 4 (Peprotech, London, UK) and 20 ng/ml FGF2 (Baxter et al., 2009). Alternatively, they were cultured feeder free, on mTESR medium (Stem Cell Technologies) on Vitronectin substrate (Thermo). The hESCs were differentiated in a basal medium (DMEM:F12, 2 mM l-glutamine, 1% (vol/vol) ITS, 1% (vol/vol) nonessential amino acids, 2% (vol/vol) B27, 90 μM β-mercaptoethanol) supplemented with appropriate sequential addition of growth factors as indicated in Fig. 1, as previously described (Oldershaw et al., 2010); Stage 1 (day 1–3): Wnt3a (25 ng/ml R&D Systems), Activin-A (reducing from 50 to 10 ng/ml, Peprotech) and BMP4 (40 ng/ml; Peprotech), followed by Stage 2 (day 4–8): BMP4 (40 ng/ml), Follistatin (100 ng/ml) and GDF5 (20 ng/ml) (all Peprotech) and finally Stage 3 (day 9–14): GDF5 (20 rising to 40 ng/ml), FGF2 (20 ng/ml) and NT4 (2 ng/ml) (all Peprotech) (Fig. 1). In some experiments BMP2 (40 ng/ml days 3–8, 20 ng/ml days 9–10) was substituted for the same concentration of BMP4 (both Peprotech), or at the concentration shown in Fig. 1. Cells were passaged on day 5 and day 8 with a change of substrate from fibronectin to fibronectin: gelatin 50:50 at day 8. For some experiments from day 11 to day 14 of the protocol, GDF5 was either replaced by BMP7 (100 or 300 ng/ml; Peprotech) together with either TGFβ1 (10 ng/ml; Peprotech) or TGFβ3 (10 ng/ml; Peprotech), or BMP7 (300 ng/ml) and TGFβ1 or TGFβ3 were added in addition to GDF5. Gene expression was analysed on days 4, 8 and 14 of the protocol.

The proliferation and apoptosis of chondrogenic cells was assessed in triplicate samples using Celltiter-Glo™ reagent and Caspase-Glo 3/7 and quantifying luminescence signal on a Glomax multidetection system (Promega).

### 2.2 Aggregate size assessment

The numbers of aggregates were counted manually in 3 wells of 6 well plates (HUES1) or 17 wells of 24 well plates (MAN7). The largest 20 aggregates for each growth factor were identified by eye and the size was measured using Image J. For gene expression analysis the aggregates were isolated manually, and RNA was collected from the aggregate cells and non-aggregate cells. cDNA was analysed by QRT-PCR.

### 2.3 Gene expression analysis

Total RNA was extracted using mirVana™ miRNA Isolation Kit (Life Technologies), reverse transcribed using M-MLV reverse transcriptase (Promega) and candidate genes expression (normalised to GAPDH) assessed using SYBR Green PCR Master Mix (Applied Biosystems) with an ABI PRISM 7500 Real Time System (Applied Biosystems). At least three separate biological repeats analysed in triplicate were performed.

### 2.4 Cell lysate fraction and western blotting

RIPA buffer cell extracts were resolved on 10% SDS gels and transferred to nitrocellulose membranes. After blocking, an appropriate antibody anti-SOX9 antibody (Abcam, 1 μg/ml), anti-GAPDH antibody (Cell Signalling, 1:2000), anti-type II collagen antibody (Abcam, 1 μg/ml or Santa Cruz), anti-SMAD1/5/9 (Abcam, 1 μg/ml) or anti-p-SMAD1/5/9 (Cell Signalling, 1 μg/ml) was added overnight followed by appropriate LI-COR secondary antibody and membranes exposed using Odyssey CLx Imager (LI-COR).

### 2.5 Immunofluorescence

Cells cultured on fibronectin coated plastic were fixed using 4% paraformaldehyde fixed and stained with primary antibody (SOX9, Millipore, 5 μg/ml) or IgG control followed by one hour in appropriate secondary antibody in blocking buffer.

### 2.6 Qualitative PCR

Samples were collected from pluripotent hESCs and at different times after the start of differentiation for analysis of *COL2A* isoforms, *COL2A1-IIa* and *COL2A1-IIb*. hESCs were cultured either with BMP4 or BMP2 from day 3–10, RNA was isolated, reverse transcribed and subject to PCR. Reaction conditions were denaturation 45 s, 94C; Annealing 45 s, 60C; elongation 45 s, 72C for 35 cycles and expression was analysed using 1.5% agarose gel electrophoresis. Primers were forward CTGCTCGTCCCGCTGTCCTT and reverse AAGGGTCCCAGGTTCTCCATC (SIGMA).

### 2.7 Flow cytometry

Single-cell suspensions were fixed in ice-cold methanol (10 min at −20 °C) and permeabilized before incubation with primary antibody (mouse anti-OCT4 [Cell Signalling], goat anti-human SOX9 [Millipore]). Flow cytometry was conducted using a BD Biosciences Fortessa and the software Diva gating was used for analysis.

### 2.8 Alkaline phosphatase assay

SW1353 cells were seeded at a density of 2.1 × 10^5^ cells in each well of 24 well plates, then treated with different concentrations of BMP2 and BMP4 for three days. Total protein was collected, and alkaline phosphatase activity was measured using fluorescence substrate 2′-[2-benzothiazoyl]-6′-hydroxybenzothiazole phosphate on a Promega Glomax multi-detection system.

### 2.9 Osteochondral defect model and histology/immunohistochemistry

Animal work had local ethical approval and was carried out under Home Office licence as required for animal husbandry and experimentation in the UK. Osteochondral defects (2 mm) were made in the trochlea groove of the femur in athymic RNU rats (Charles River, Germany) as previously described (Cheng et al., 2014b). Fibrin constructs were formed by resuspending 3 × 10^6^ cells in 50 μl fibrinogen followed by adding 50 μl thrombin and implants (containing 2 × 10^5^ cells) were cut to fit the defects using a biopsy punch (Cheng et al., 2014a). Defects without implant or with cell-free fibrin gel served as controls. Joints were fixed in 10% neutral buffered formalin, decalcified and embedded in paraffin for histology. Histological scoring for cartilage repair by 3 independent observers was by Pineda's method (Pineda et al., 1992; Orth et al., 2012).

### 2.10 Statistics

Data are shown as the mean ± SEM. An unpaired *t*-test for data from two groups, or one-way ANOVA for data from groups of three, or more was used to compare data with post hoc Tukey's test for gene expression comparison and Bonferroni's test for histology analysis. P values < 0.05 were considered to be significant.

## 3 Results

### 3.1 Assessment of the effects of different TGFβ family growth factors in the chondrogenic differentiation protocol

**Fig. 2:**
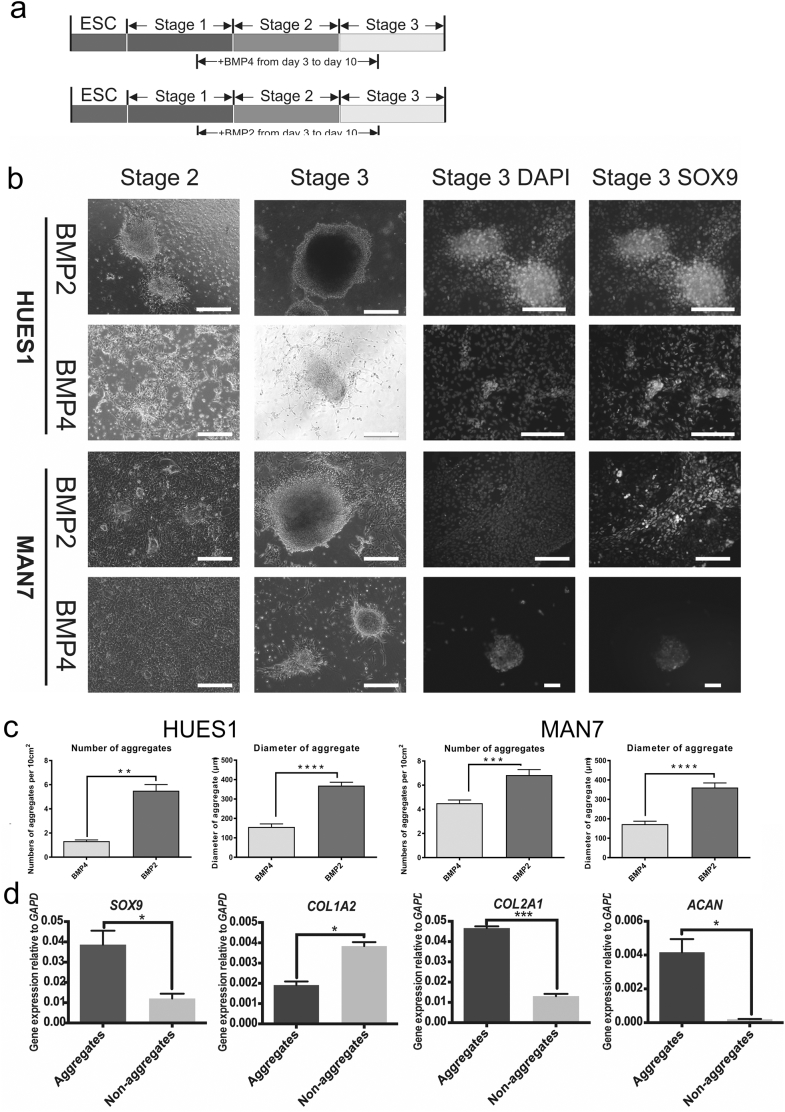
Effects of BMP2 substitution for BMP4 from day 3–10 of the chondrogenic protocol. (a) The protocol overview of BMP2 and BMP4 treatments to induce chondrogenesis. (b) Cell morphology was observed under phase contrast microscopy. The chondrogenic protocol was carried out as previously described and cells were observed on day 8 and day 14. In the right panel, cells were stained with anti-SOX9 antibody for immunofluorescence. Scale bar 100 μm. (c) The comparison of number and size of aggregates between BMP2 and BMP4 treatment on HUES1 and MAN7. All data are shown as mean ± SEM. Student's *t*-test was performed. ^⁎⁎^P < 0.01, ^⁎⁎⁎⁎^P < 0.0001. (d) QRT-PCR gene transcription analysis of aggregates and non-aggregate cells formed during the BMP2 driven chondrogenic differentiation of MAN7 cells All data are shown as relative expression compared to GAPDH + SEM (n = 3). Student's t-test was performed. ^⁎^P < 0.05, ^⁎⁎⁎^P < 0.0005, ^⁎⁎⁎⁎^P < 0.0001.

**Fig. 3:**
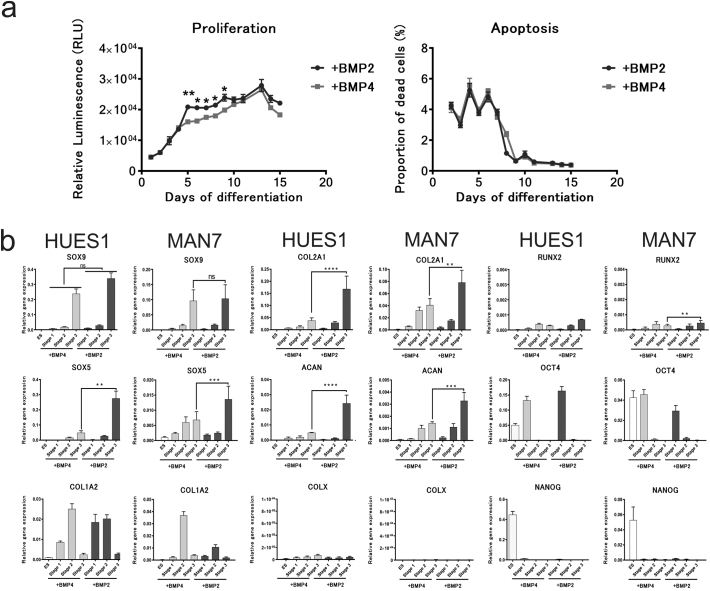
Effects of BMP2 substitution for BMP4 from day 3–10 on proliferation/apoptosis and chondrogenic associated genes expression during the chondrogenic protocol. (a) Proliferation and apoptosis during chondrogenesis with BMP2, (green) or BMP4, (red) from day 3–10 of the protocol on HUES1 cells. Data shown as mean ± SEM. One way-ANOVA was performed. ^⁎⁎⁎^ = P < 0.005. (b) Gene expression of chondrogenic and pluripotency associated genes after BMP4 or BMP2 treatment on HUES1 and MAN7 cells. All data are shown as relative expression compared to GAPDH (or ACTB) + SEM (n = 3). One way-ANOVA was performed, and Tukey's test was performed as post-hoc analysis. ^⁎⁎⁎^P < 0.0005. ^⁎⁎⁎⁎^P < 0.0001. (For interpretation of the references to colour in this figure legend, the reader is referred to the web version of this article.)

**Supplementary Fig. S1:**
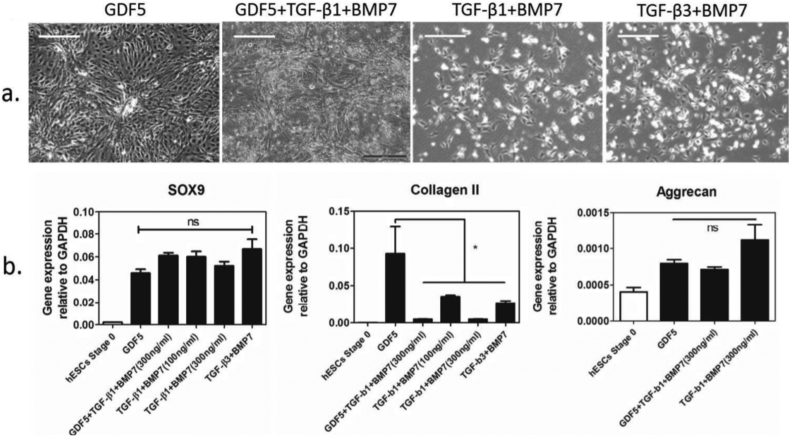
Effects of different growth factors in stage 3 of chondrogenic protocol in HUES1. Chondrogenic protocol was carried out as described before until day 10, from day 11 to day 13 cells were treated with different combination of growth factors and on day 14 cell morphology was observed and photographed under phase contrast (a). RNA was collected on day 14 for gene expression analysis (b).

With the aim of generating more mature chondrogenic cells with a greater capacity to produce matrix in vitro, we looked at the effect of BMP7, TGFβ-1 and TGFβ-3 as alternatives to, or in addition to, GDF5, in stage 3 of our protocol (day 9–14). Cells cultured with GDF5 showed typical chondrogenic cell morphology and produced cell aggregates, but cells cultured without GDF5, but with BMP7 together with either TGFβ-1 or TGFβ-3 showed pronounced cell death. In addition, QRT-PCR showed that the removal, or replacement of GDF5 in the protocol caused a dramatic decrease in *COL2A1* expression, without affecting the expression of *SOX9* (Supplementary Fig. S1). When we added BMP7, together with either TGFβ-1, or TGFβ-3 (GDF5 still present) there was less cell death, but there was a similar reduction in *COL2A1* with no change in *SOX9* or *ACAN* expression (Supplementary Fig. S1). Although BMP4 has been shown to be most active in early mesodermal patterning in mice, BMP2 has been shown in different chondrogenic systems to have strong pro-chondrogenic effects (Toh et al., 2005; Duprez et al., 1996). We therefore tested the substitution of BMP4, used from day 3 to 10 to induce mesoderm and later chondrogenic mesoderm, with BMP2 (Figs. 2 and 3). Results using 2 hESC lines of different origin (HUES1 and MAN7), both showed that with BMP2 there was earlier appearance of cell aggregates during stage 2, which typified chondrogenic differentiation, whereas they were delayed until stage 3 in the protocol with BMP4. Moreover, with BMP2 the aggregates were significantly larger (Fig. 2c), although there was no significant change in overall cell expansion, or apoptosis (Fig. 3). In the cultures under the conditions tested, there was an approximately 8-fold increase in cell number by day 10 in the presence of either BMP2 or 4, which was as reported previously (Oldershaw et al., 2010). However, during the protocol there was a small but significant increase in cell number with BMP2 compared to BMP4 between day 5 and day 9 (Fig. 3), suggesting an early increase in proliferation. At the end of the protocol the cells in aggregates were manually separated from those in between aggregates and they were assessed separately for chondrogenic gene expression. This confirmed that the cells in aggregates were enriched in the chondrogenic transcripts *COL2A1*, *SOX9* and *ACAN,* while cells outside aggregates had higher expression of collagen *COL1A1,* which is more associated with non-chondrogenic fibroblastic cells (Fig. 2d).

Comparing gene expression between BMP2 and 4 induced cells (Fig. 3) revealed that the pluripotency-associated markers, *OCT4* and *NANOG,* decreased early in both cultures. They also both showed similar *SOX9* expression, but the *SOX9* cofactor *SOX5* was significantly increased at day 14 in BMP2 cultures. The strong chondrogenic drive of BMP2 was revealed most markedly by major increases in the expression of both *COL2A1* and *ACAN,* which were increased by 4 and 8.5-fold respectively in the presence of BMP2. This chondrogenic enhancement was also supported by a 2-fold decrease in the expression of *COL1A1*. There was also no change in *COL10* expression, which was very low in both BMP2 and BMP4 cultures. The effects of BMP2 were similar in both hES cell lines tested.

**Fig. 4:**
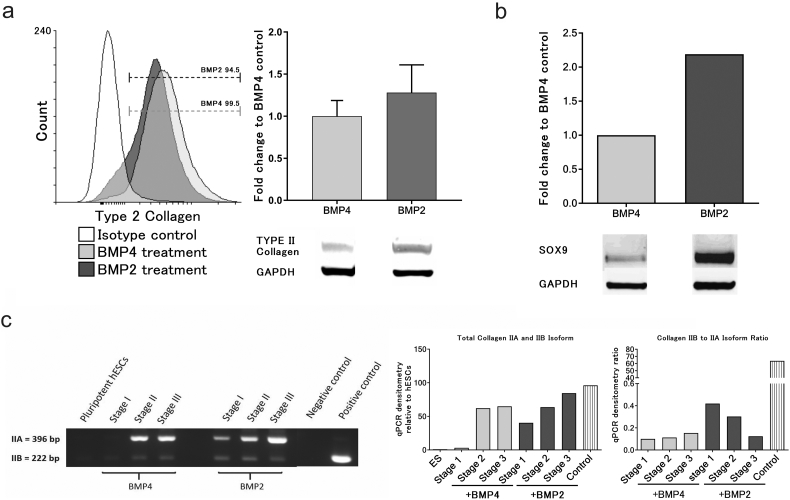
Effects of BMP2 substitution for BMP4 from day 3–10 on chondrogenic associated protein expression and maturation of chondrogenic cells during the protocol. (a) The protein expression of type II collagen after BMP4 or BMP2 treatment on chondrogenic cells generated from MAN7 (n = 5), and (b) expression of SOX9. Chondrogenic cells on day14 were treated with protein transport inhibitor cocktail for 6 h, with protein analysed by western blot, and the quantitative protein expression data displayed relative to GAPDH. (c) The qualitative RT-PCR of collagen 2A1 IIA and IIB isoform expressed during chondrogenic differentiation with either BMP2 or BMP4. A positive control was obtained from human tibial cartilage.

**Supplementary Fig. S2:**
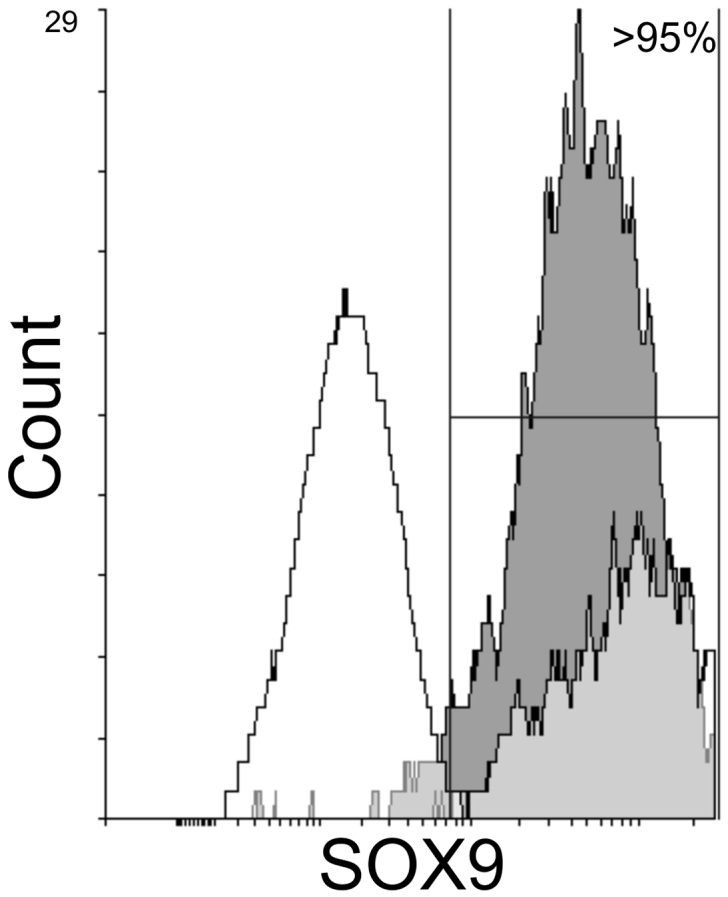
FACS blot for SOX9 labelled cells differentiated with either BMP2 (dark grey) or BMP4 (light grey) in the chondroprogenitor differentiation protocol.

Since BMP2 increased the expression of genes associated with chondrogenesis we validated the effect by measuring proteins expression in hESC chondrogenic cells at the end of stage 3. Collagen produced by the cells is constitutively secreted into the extracellular matrix, we therefore used a protein transport inhibitor cocktail (Brefeldin A and Monensin) for 6 h prior to collecting cell lysate in order to compare cellular Collagen in the 2 cultures at day 14. In both BMP4 and BMP2 cell lysates a strong type II collagen protein band was detected (Fig. 4a). The BMP2 cultured cells showed a stronger band for SOX9 protein compared to BMP4 cells at the end of stage 3 (Fig. 4b). Parallel flow cytometry showed >95% of cells were SOX9 positive at the end of stage 3 in both BMP4 and BMP2 cultures (Supplementary Fig. S2).

Since mature chondrocytes produce an alternatively spliced transcript, *COL2A1* IIB, in which exon 2 is omitted, while in prechondrogenic mesoderm the prevalent form is IIA, in which exon 2 is retained (Ryan and Sandell, 1990; McAlinden, 2014) we measured transcript expression to detect if this transition occurred in our cultures. The analysis of splice-form specific transcripts showed that in both BMP2 and 4 cultures the predominant isoform at day 14 was IIA, but a weak band for IIB was also detected (Fig. 4c). Interestingly in this analysis the expression of both isoforms of *COL2A1* but notably IIb occurred earlier in the BMP2 cultured cells than in BMP4 cultures.

**Fig. 5:**
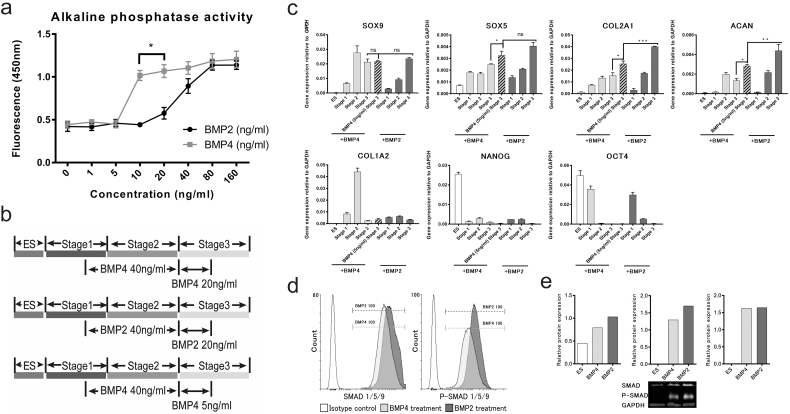
BMP2 had lower biological activity but gave higher chondrogenic associated gene expression and phosphorylation of SMAD proteins in hESC chondrogenesis. (a) Alkaline phosphatase assay of SW1353 cells, were treated with different concentrations of BMP2 and BMP4 for three days. (b) Protocol overview of BMP2 and BMP4 treatments to induce chondrogenesis. (c) QRT-PCR gene expression analysis of chondrogenic and pluripotency associated genes after BMP4 or BMP2 treatment on MAN7 cells. All data are shown as relative expression compared to GAPDH mean ± SEM (n = 3). One way-ANOVA was performed. ^⁎⁎⁎^P < 0.0005. ^⁎⁎⁎⁎^P < 0.0001. (d) Representative flow cytometry data for protein expression of SMAD1/5/9 and p-SMAD1/5/9 after BMP4 or BMP2 treatment, on chondrogenic cells generated from MAN7. (e) Western blot analysis of SMAD1/5/9 and p-SMAD1/5/9 in day10 cells treated with BMP2 or BMP4 for one-hour. Protein expression shown as relative to GAPDH.

BMP2 and BMP4 are often used interchangeably in research and bind to the same receptors. The above results suggest that BMP2 generates more chondrogenic signals in the hESC cultures, than BMP4. This may result from BMP2 synergising with additional co-factors creating higher BMP2 potency. To assay activity independently, we measured the induction of alkaline phosphatase in the chondrosarcoma line SW1353 (Fig. 5a). The results suggested that intrinsically BMP4 was *more* potent than BMP2 at 10-20 ng/ml in SW 1353 chondrosarcoma cells. Although this may not hold for developing chondrogenic cells this result suggests that the greater effect of BMP2 on hESCs was unlikely to be due to a simple difference in its potency.

### 3.2 SMAD signalling after BMP2 and 4 treatment

We next examined if signalling from the two BMPs was similar (Fig. 5d). BMP binding to its heterodimeric receptor activates phosphorylation of SMAD1, 5 and 9 which further interact with co-SMADs and after translocation to the nucleus drive changes in gene transcription. Flow cytometry analysis of day-14 cells, showed no significant difference between BMP4 and BMP2 treatments in the number of cells positive for SMAD1/5/9 and p-SMAD1/5/9. This suggested that receptor signal transduction through the SMAD pathway from BMP2 and 4 was similar in day 14 hESC chondroprogenitors. Confirming this, when the ratio of p-SMAD1/5/9 to total SMAD1/5/9 was normalised to GAPDH in extracts from cells examined on day 10, there was no difference between BMP2 and BMP4 (both at 20 ng/ml, Fig. 5e).

### 3.3 Does BMP2 induction of hESC-chondroprogenitors result in improved cartilage repair in vivo?

**Fig. 6:**
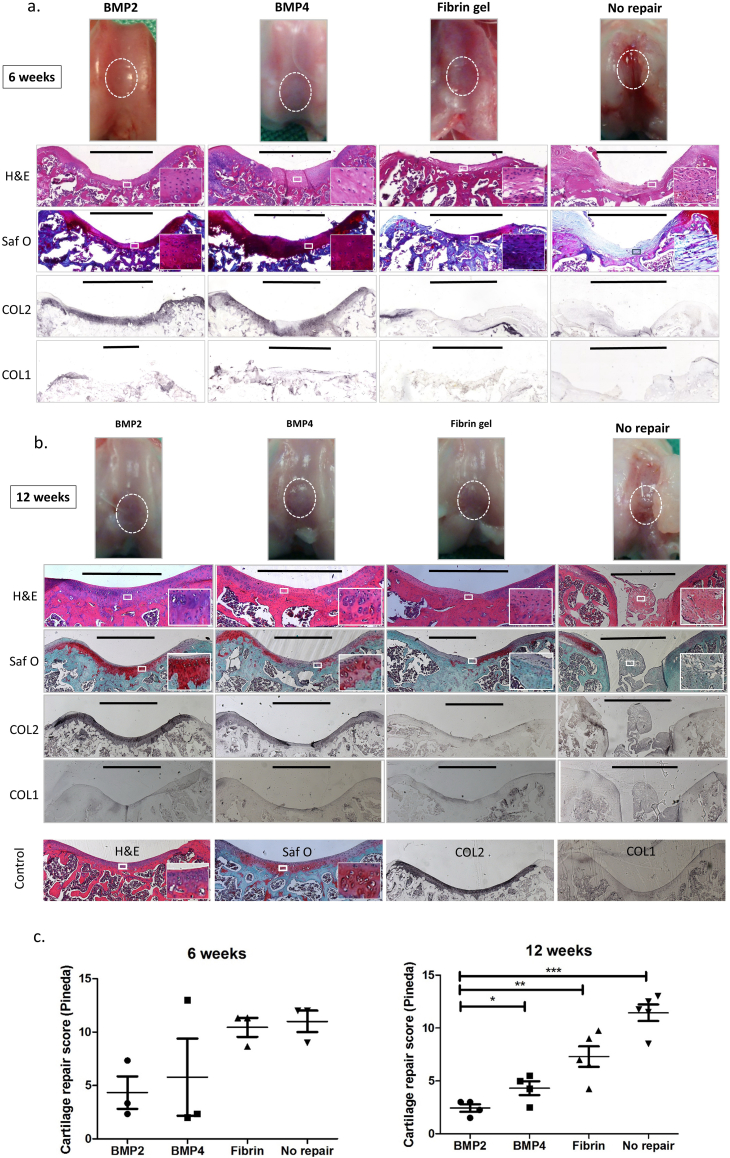
In vivo osteochondral repair in athymic RNU rats using chondroprogenitors derived from hESCs cultured with BMP2, or BMP4 or without chondroprogenitors (fibrin gel alone, or defects left unfilled). Gross assessment and coronal plane histological analysis were carried out 6 weeks (a) and 12 weeks (b) after the surgery. Boxes show expanded area. Bars indicate the width of osteochondral defects and of the cartilage repair. The cartilage repair score (Pineda's system) after the osteochondral defect +/− repair in athymic RNU rats was used for evaluation after 6 and 12 weeks (c). The score ranges from 0 (best) to 14 (worst). ^⁎^ = P < 0.05, ^⁎⁎^ = P < 0.01, ^⁎⁎⁎^ = P < 0.001.

To test whether fibrin gels containing BMP2 induced progenitors promote improved cartilage repair in vivo we embedded hESC-chondrogenic cells from day 14 in fibrin matrix and implanted them in 2 mm diameter osteochondral defects in the trochlea groove of the femur of Nude rats as previously described (Cheng et al., 2014b). Control defects were left empty or repaired with fibrin gel alone, and repair was analysed at 6 and 12 weeks (Fig. 6). Two animals were removed from the study because in each case dislocation of the patella had occurred during joint surgery. The empty control defects retained a marked cavity and showed no evidence of organised repair by 12 weeks. When fibrin alone was used, repair with a more uniform surface was generated, but with little type II collagen immunostaining and only a few isolated areas of Safranin O staining. In contrast, in the defects repaired with cells embedded in fibrin, there was strong type II collagen immunostaining and Safranin O positive areas and there was also good integration with the surrounding tissue. Cartilage repair was assessed in sections using the system developed by Pineda (Pineda et al., 1992). The repair associated with the fibrin gels containing cells induced with BMP2 appeared better than those with BMP4 cells and when the sections were scored blind by 3 observers a significant improvement was detected with BMP2 cells.

## 4 Discussion

TGFβ-family growth factors play roles during mesenchymal condensation and cartilage formation, (Cheng et al., 2014b; Steinert et al., 2003). Therefore, we examined the effect of TGFβ-1, TGFβ-3 and BMP7 on the differentiation protocol in enhancing the hESC-chondrogenic phenotype. We found that replacing GDF5 with BMP7 together with TGFβ-1 or -β3 from day 11 produced no improvement in *SOX9* expression and reduced *COL2A1* expression. This is in distinction to Nakagawa et al. (2009) who reported chondrocyte formation from hESCs in the presence of BMP7 and TGFβ-1, albeit under a less defined culture regimen including serum. In the absence of GDF5, which in other tissues can function as a survival factor (Jaumotte and Zigmond, 2014; Sullivan et al., 1998), hESCs showed pronounced apoptosis. This suggests that GDF5 has a survival effect on human chondrogenic cells, especially since adding GDF5 in the presence of BMP7 and TGFβ-1 suppressed cell death. However, in the BMP7/TGFβ-1/GDF5 protocol, these growth factors did not enhance chondrogenesis, as there was a decrease in expression of *COL2A1* and no significant increases in *ACAN*, or *SOX9*. The specific role of GDF5 in maturation of the chondrocytes and establishment of the joint hyaline cartilage is difficult to assess, because of the loss of cells in its absence. However, as well as a role in enhancing cartilage formation (Hatakeyama et al., 2004; Thomas et al., 1996; Thomas et al., 2006; Roelofs et al., 2017; Shwartz et al., 2016), GDF5 is suggested to play a role in modulation of BMP action during the formation of the joint and the interzone and at a time when permanent cartilage is well established (Tsumaki et al., 2002; Thomas et al., 2006).

In contrast, substituting BMP2 for BMP4 from day 3 led to enhanced expression of chondrogenic transcription factors and importantly increased *COL2A1* and *ACAN* expression at the end of the protocol in both hESC lines examined. We confirmed that cell aggregation reflected an enriched chondrogenic cell population with increased *COL2A1, ACAN* and *SOX5* expression and the size of the condensations formed was increased with BMP2, suggesting a larger proportion of chondrogenic cells. Although initial proliferation was slightly greater in the presence of BMP2, confirming the published effect on proliferation (Shu et al., 2011), final culture expansion in the protocol remained the same for both BMPs and there was no evidence of differing apoptosis. Although *COL2A1* and *ACAN* expression were substantially increased in BMP2 cultures, there was no significant change in the expression of *SOX9*, but there was increased expression of *SOX5,* which acts co-ordinately with SOX9 in driving matrix gene expression and may have been a factor limiting the transcription of the *COL2A1* gene.

BMP2 and 4 have often been used interchangeably in experiments in cell and developmental systems. Therefore, it was surprising that using BMP2 at the same concentration as BMP4 showed such a pronounced effect on matrix gene expression. The two growth factors have high structural homology, they are the most closely related of the BMP-subfamily and interact with the same receptors (BMPR1a, b [ALK3, ALK6], BMPR2 [ALK4]). However, although Flow cytometry and immunoblotting showed little difference in SMAD signalling between BMP2 and BMP4 derived cells, additional signal pathways may be triggered by BMP2 resulting from effects on receptor lateral mobility and clustering (Guzman et al., 2012). Analysis showed that by the end of the protocol SOX9 protein was increased in BMP2 cultured cells compared to BMP4. This may be a consequence of additional signals from BMP2 resulting in increased cell rounding and cytoskeletal relaxation inherent in cell aggregate formation, which is known to promote chondrocyte differentiation (Woods et al., 2005) and has been reported to increase the half-life of SOX9 transcripts in chondrocytes (Tew and Hardingham, 2006). In our cultures there was no large increase in SOX9 transcript levels in BMP2 cultures, but it is possible that there are additional direct effects on protein translation through the cell shape and cytoskeletal changes accompanying aggregate formation.

The differential effects of BMP2 and BMP4 may therefore be modulated by their different interactions with extracellular matrix components that regulate their association and clustering with receptors at the cell surface (Sieber et al., 2009). This is likely to be governed by the rapid changes in matrix components during differentiation. Although BMP2 is established as involved in osteogenic differentiation (Shu et al., 2011), we saw no evidence of COLX expression during differentiation and it is clear that during early development BMP2 has roles in limb development (Duprez et al., 1996) initiation of chondrogenesis, chondrocyte proliferation (Bandyopadhyay et al., 2006) and stimulates synthesis of cartilage collagen II and proteoglycans including aggrecan (Blaney Davidson et al., 2007).

In order to assess any benefit of BMP2 in the differentiation protocol we tested the repair potential of BMP2 and BMP4 hESCs in vivo. The results showed that implanting fibrin gels containing BMP2 and BMP4 induced-cells produced effective tissue repair significantly better than with cell-free fibrin implants. The histological scoring showed that BMP2 induced cells produced significantly better quality of repair at 12 weeks compared to BMP4 induced cells. In previous work (Cheng et al 2014b) we have shown that hESC-derived chondrocytes can be found in the implant area at 8 or 12 weeks after implantation suggesting that they contribute to the repair process. This current work therefore suggests that BMP2 generated chondroprogenitors support better cartilage matrix repair in vivo compared to those generated by BMP4.

Our protocol provides an experimental in vitro system permitting detailed analysis of the mechanisms driving human chondrogenesis in development and in tissue repair. For the latter we are now applying our refined protocol to clinical grade hESCs, which, would be suitable for repairing human cartilage defects. The procedure we developed also provides a platform for studying early development and joint disease mechanisms and by using iPSCs from patients it enables the study of genetic diseases affecting joint cartilage.

The following are the supplementary data related to this article.

## Data avaliability statement

The data that support the findings of this study are available from the corresponding author upon reasonable request.

## References

[bb0005] Bandyopadhyay A., Tsuji K., Cox K. (2006). Genetic analysis of the roles of BMP2, BMP4, and BMP7 in limb patterning and skeletogenesis. PLoS Genet..

[bb0010] Baxter M.A., Camarasa M.V., Bates N. (2009). Analysis of the distinct functions of growth factors and tissue culture substrates necessary for the long-term self-renewal of human embryonic stem cell lines. Stem Cell Res..

[bb0015] Blaney Davidson E.N., Vitters E.L., van Lent P.L. (2007). Elevated extracellular matrix production and degradation upon bone morphogenetic protein-2 (BMP-2) stimulation point toward a role for BMP-2 in cartilage repair and remodeling. Arthritis Res. Ther..

[bb0020] Brittberg M., Lindahl A., Nilsson A. (1994). Treatment of deep cartilage defects in the knee with autologous chondrocyte transplantation. N. Engl. J. Med..

[bb0025] Cheng A., Hardingham T.E., Kimber S.J. (2014). Generating cartilage repair from pluripotent stem cells. Tissue Eng. B Rev..

[bb0030] Cheng A., Kapacee Z., Peng J. (2014). Cartilage repair using human embryonic stem cell-derived chondroprogenitors. Stem Cells Transl. Med..

[bb0035] Craft A.M., Rockel J.S., Nartiss Y. (2015). Generation of articular chondrocytes from human pluripotent stem cells. Nat. Biotechnol..

[bb0040] Diekman B.O., Christoforou N., Willard V.P. (2012). Cartilage tissue engineering using differentiated and purified induced pluripotent stem cells. Proc. Natl. Acad. Sci. U. S. A..

[bb0045] Duprez D.M., Coltey M., Amthor H. (1996). Bone morphogenetic protein-2 (BMP-2) inhibits muscle development and promotes cartilage formation in chick limb bud cultures. Dev. Biol..

[bb0050] Filardo G., Kon E., Berruto M. (2012). Arthroscopic second generation autologous chondrocytes implantation associated with bone grafting for the treatment of knee osteochondritis dissecans: results at 6 years. Knee.

[bb0055] Francis-West P.H., Parish J., Lee K. (1999). BMP/GDF-signalling interactions during synovial joint development. Cell Tissue Res..

[bb0060] Gaissmaier C., Koh J.L., Weise K. (2008). Growth and differentiation factors for cartilage healing and repair. Injury.

[bb0065] Guzman A., Zelman-Femiak M., Boergermann J.H. (2012). SMAD versus non-SMAD signaling is determined by lateral mobility of bone morphogenetic protein (BMP) receptors. J. Biol. Chem..

[bb0070] Hatakeyama Y., Tuan R.S., Shum L. (2004). Distinct functions of BMP4 and GDF5 in the regulation of chondrogenesis. J. Cell. Biochem..

[bb0075] Hettrich C.M., Crawford D., Rodeo S.A. (2008). Cartilage repair: third-generation cell-based technologies—basic science, surgical techniques, clinical outcomes. Sports Med. Arthrosc. Rev..

[bb0080] Jaumotte J.D., Zigmond M.J. (2014). Comparison of GDF5 and GDNF as neuroprotective factors for postnatal dopamine neurons in ventral mesencephalic cultures. J. Neurosci. Res..

[bb0085] Kang S.W., Yoo S.P., Kim B.S. (2007). Effect of chondrocyte passage number on histological aspects of tissue-engineered cartilage. Biomed. Mater. Eng..

[bb0090] Laflamme M.A., Chen K.Y., Naumova A.V. (2007). Cardiomyocytes derived from human embryonic stem cells in pro-survival factors enhance function of infarcted rat hearts. Nat. Biotechnol..

[bb0095] Lee J.S., Ha L., Kwon I.K. (2013). The role of focal adhesion kinase in BMP4 induction of mesenchymal stem cell adipogenesis. Biochem. Biophys. Res. Commun..

[bb0100] McAlinden A. (2014). Alternative splicing of type II procollagen: IIB or not IIB?. Connect. Tissue Res..

[bb0105] Nakagawa T., Lee S.Y., Reddi A.H. (2009). Induction of chondrogenesis from human embryonic stem cells without embryoid body formation by bone morphogenetic protein 7 and transforming growth factor beta1. Arthritis Rheum..

[bb0110] Oldershaw R.A., Baxter M.A., Lowe E.T. (2010). Directed differentiation of human embryonic stem cells toward chondrocytes. Nat. Biotechnol..

[bb0120] Orth P., Zurakowski D., Wincheringer D. (2012). Reliability, reproducibility, and validation of five major histological scoring systems for experimental articular cartilage repair in the rabbit model. Tissue Eng. C Methods.

[bb0125] Pelttari K., Winter A., Steck E. (2006). Premature induction of hypertrophy during in vitro chondrogenesis of human mesenchymal stem cells correlates with calcification and vascular invasion after ectopic transplantation in SCID mice. Arthritis Rheum..

[bb0130] Pineda S., Pollack A., Stevenson S. (1992). A semiquantitative scale for histologic grading of articular cartilage repair. Acta Anat..

[bb0135] Pittenger M.F., Mackay A.M., Beck S.C. (1999). Multilineage potential of adult human mesenchymal stem cells. Science.

[bb0140] Roelofs A.J., Zupan J., Riemen A.H.K. (2017). Joint morphogenetic cells in the adult mammalian synovium. Nat. Commun..

[bb0145] Ryan M.C., Sandell L.J. (1990). Differential expression of a cysteine-rich domain in the amino-terminal propeptide of type II (cartilage) procollagen by alternative splicing of mRNA. J. Biol. Chem..

[bb0150] Shu B., Zhang M., Xie R. (2011). BMP2, but not BMP4, is crucial for chondrocyte proliferation and maturation during endochondral bone development. J. Cell Sci..

[bb0155] Shwartz Y., Viukov S., Krief S. (2016). Joint development involves a continuous influx of Gdf5-positive cells. Cell Rep..

[bb0160] Sieber C., Kopf J., Hiepen C. (2009). Recent advances in BMP receptor signaling. Cytokine Growth Factor Rev..

[bb0165] Steinert A., Weber M., Dimmler A. (2003). Chondrogenic differentiation of mesenchymal progenitor cells encapsulated in ultrahigh-viscosity alginate. J. Orthop. Res. Off. Publ. Orthop. Res. Soc..

[bb0170] Stolzing A., Jones E., McGonagle D. (2008). Age-related changes in human bone marrow-derived mesenchymal stem cells: consequences for cell therapies. Mech. Ageing Dev..

[bb0175] Sullivan A.M., Pohl J., Blunt S.B. (1998). Growth/differentiation factor 5 and glial cell line-derived neurotrophic factor enhance survival and function of dopaminergic grafts in a rat model of Parkinson's disease. Eur. J. Neurosci..

[bb0180] Tew S.R., Hardingham T.E. (2006). Regulation of SOX9 mRNA in human articular chondrocytes involving p38 MAPK activation and mRNA stabilization. J. Biol. Chem..

[bb0185] Thomas J.T., Lin K., Nandedkar M. (1996). A human chondrodysplasia due to a mutation in a TGF-beta superfamily member. Nat. Genet..

[bb0190] Thomas J.T., Prakash D., Weih K. (2006). CDMP1/GDF5 has specific processing requirements that restrict its action to joint surfaces. J. Biol. Chem..

[bb0195] Toh W.S., Liu H., Heng B.C. (2005). Combined effects of TGFbeta1 and BMP2 in serum-free chondrogenic differentiation of mesenchymal stem cells induced hyaline-like cartilage formation. Growth Factors.

[bb0200] Tsumaki N., Nakase T., Miyaji T. (2002). Bone morphogenetic protein signals are required for cartilage formation and differently regulate joint development during skeletogenesis. J. Bone Miner. Res..

[bb0205] Wakitani S., Okabe T., Horibe S. (2011). Safety of autologous bone marrow-derived mesenchymal stem cell transplantation for cartilage repair in 41 patients with 45 joints followed for up to 11 years and 5 months. J. Tissue Eng. Regen. Med..

[bb0210] Winnier G., Blessing M., Labosky P.A. (1995). Bone morphogenetic protein-4 is required for mesoderm formation and patterning in the mouse. Genes Dev..

[bb0215] Woods A., Wang G., Beier F. (2005). RhoA/ROCK signaling regulates Sox9 expression and actin organization during chondrogenesis. J. Biol. Chem..

[bb0220] Xu R.H., Chen X., Li D.S. (2002). BMP4 initiates human embryonic stem cell differentiation to trophoblast. Nat. Biotechnol..

[bb0225] Yamashita A., Morioka M., Yahara Y. (2015). Generation of scaffoldless hyaline cartilaginous tissue from human iPSCs. Stem Cell Rep..

[bb0230] Zhang P., Li J., Tan Z. (2008). Short-term BMP-4 treatment initiates mesoderm induction in human embryonic stem cells. Blood.

[bb0235] Zou H., Choe K.M., Lu Y. (1997). BMP signaling and vertebrate limb development. Cold Spring Harbor Symposia on Quantitative Biology.

